# Assessing the Awareness and Perception of Telemedicine Among the General Population in the Al-Baha Region, Saudi Arabia

**DOI:** 10.7759/cureus.49732

**Published:** 2023-11-30

**Authors:** Terad A Talmesany, Meshal A Alzahrani, Omran M Alzahrani, Sultan A Alzahrani, Tahani K Al-Omari, Sumaeah M Alghamdi, Mohammed A Alzahrani

**Affiliations:** 1 Community and Family Medicine, Faculty of Medicine, Al-Baha University, Al-Baha, SAU; 2 General Practice, Prince Meshari Bin Saud Hospital, Al-Baha, SAU; 3 General Practice, King Fahad Hospital, Al-Baha, SAU; 4 Medicine, Faculty of Medicine, Al-Baha University, Al-Baha, SAU

**Keywords:** quality, ict, technology, healthcare, telemedicine

## Abstract

Telemedicine plays an important role in healthcare by improving the quality of the healthcare system. However, various challenges associated with the effective implementation of telemedicine have been reported. This study aimed to explore the awareness and utilization of telemedicine services among the general population in the Al-Baha region of Saudi Arabia and the factors affecting it. Using a cross-sectional design for the study, the quantitative approach was employed through a questionnaire-based survey. Data from 359 participants from the Al-Baha general population were collected, including both males and females over the age of 18. The analysis of the collected data shows a low level of familiarity within the general population; indeed, 54.9% (197) of participants have experienced using telemedicine services. Moreover, the study reveals that the major concerns influencing the use of telemedicine services are limited availability, privacy and security, and quality of care. Almost half of the participants have used telemedicine, and they expressed concerns related to quality of care, privacy and security, limited availability, and technical difficulties. However, telemedicine was positively perceived among the participants. There is a need to raise public awareness about the importance and effectiveness of telemedicine.

## Introduction

Background

Over the past year, there have been extraordinary developments in the field of technology. More specifically, information and communication technology (ICT) has witnessed tremendous growth in almost every sector of the economy. ICT helps governments, businesses, and citizens increase the efficiency of communication and enhance their capability to construct informed decisions that contribute to a knowledge-based economy [1]. It has had significant influence in almost every sector of the economy, from education to business and the overarching institutional framework. In the same way, ICT has also had a profound influence on the medical industry. With its great potential to support rehabilitation and its benefits and implications for clinical practice, ICT has demonstrated its uses in various areas of healthcare support [2]. Within these areas, telemedicine is considered to have enacted a major positive change in the field of medicine, working by electronically transmitting medical data from one location to another with the goal of enhancing patient health and enabling long-distance patient care and maintenance of patient health records [3]. Moreover, it is one of the methods that enable healthcare professionals to contact remote patients to offer them diagnostic and therapeutic services as a result of the rapid advancement in communications technology [4].

Various studies discuss telemedicine from a range of angles, including the telemedicine practices and associated challenges, implementation, benefits and drawbacks, and the perception of both health practitioners and the general public. Ghaddar et al. [5] examine the factors affecting the utilization and influence of telehealth services in Hispanic communities through an interview approach. The findings showed that a positive attitude toward telehealth influences the intention to use it, while the intention to use telehealth is associated with electronic health literacy. In a similar study, Wernhart et al. [6] examined the difference in the utilization of telemedicine among current and future healthcare professionals through an online survey, finding that professionals express higher levels of understanding of eHealth and telemedicine concepts compared to students. Their study also highlights that professionals have concerns regarding the effect of telemedicine use on the doctor-patient relationship and on data security and privacy issues. In a recent study, Fouad et al. [7] reported that there was a positive attitude toward technology among mental healthcare providers, but a lower level of skills, awareness, and knowledge. Furthermore, there was a significant correlation between skill level, awareness, and knowledge of telemedicine.

In the context of Saudi Arabia, Albarrak et al. [8] examined the knowledge, willingness, and perception of telemedicine among physicians in the Riyadh region, finding a low level of knowledge regarding telemedicine technologies, but a positive overall perception and willingness among physicians to use telemedicine. Similarly, Alnajrani et al. [9] documented positive perception and a high level of willingness among the general public to embrace the integration of telepharmacy services. The findings also showed a correlation between the association of knowledge and willingness of the participants and their gender. According to Mubaraki et al. [10], among 25 physicians from Taif, 36% (9) acknowledge the effectiveness of telemedicine for improving therapeutic intervention, while 44% (11) believe that telemedicine has improved the quality of care. Overall, numerous pieces of literature explore the effectiveness of telemedicine in various settings. However, several other concerns are reported, including privacy, lack of training, cost, and problems with ICT, all of which affect the adoption of telemedicine [8].

According to El Kheir et al. [11], out of 220 eligible physicians from King Fahad Hospital of the University (KFHU), only two-thirds of physicians utilize smart gadgets to administer healthcare; meanwhile, 1 in 13 reported legal issues with regulations, indicating that only a small segment of the physicians are following the telemedicine regulating guidelines. In Saudi Arabia, although authorities have implemented various strategies and investments to improve the healthcare system in terms of telehealth, various cultural and technical issues regarding telemedicine have been reported [12]. Similarly, other barriers include cultural, economic, organizational, individual, technological, legal, and regulatory issues that affect the development of the use of telemedicine [13]. Additionally, studies have shown a low level of knowledge regarding telemedicine among physicians [8]. The demand for proficient and advanced healthcare has been increasing; however, studies exist that assess the barriers relating to this area of study from the point of view of the general public. Therefore, this study will explore the awareness of the population regarding telemedicine in the Al-Baha region, specifically.

Research objectives and questions

The primary objective of the research was to explore the awareness and perception of telemedicine services among the general population in the Al-Baha region, Saudi Arabia.

Significance of the research

Telemedicine provides many beneficial outcomes in terms of decision-making, diagnostic testing, training staff, and data handling and recording. Therefore, evaluating the general public’s receptiveness to and awareness of telemedicine would provide an effective way to promote acceptance and improve the quality and safety of telemedicine practice. The study will help the relevant authorities invest effectively in health literacy and promotion to increase public awareness of the welfare of telemedicine services in the region. The study’s findings will help the relevant bodies understand the effect of sociodemographic characteristics on satisfaction with telemedicine; therefore, they will be able to target a specific segment of the population to promote a more effective healthcare system. Overall, the understating of the general public in terms of perception and satisfaction will provide valuable data to help optimize the implementation of telemedicine, thereby addressing the prevailing and increasing healthcare demands in the region.

## Materials and methods

Study design

To explore the awareness and utilization of telemedicine services among the general population in the Al-Baha region, a cross-sectional design has been selected. This research design allows the collection of a large set of data to provide a deeper understanding of the research objectives [14]. This is the most suitable research structure for investigating the awareness and perception of telemedicine among the general population, as it provides more efficient options for generalization from the study’s outcome. Moreover, the design is considered more cost-effective and less time-consuming than other research methods, being easier, quicker, and more economical while still allowing for the evaluation of prevalence [15]. Within this structure, the study also utilized a quantitative approach, with primary data collected through a questionnaire survey.

Study population and sample

Since the study aims to investigate the general population from the Al-Baha Region, data were gathered from both males and females. To select the sample from this population, the convenience sampling method was utilized, which is a useful sampling technique that allows the selection of the sample based on the availability of the participants or at the convenience of the researcher [16]. Therefore, the sample for this study was selected based on meeting the inclusion criteria, inclusive of the Al-Baha population, both male and female, aged over 18 and possessing an interest in joining our study; and the exclusive criterion being the population who are not from the Al-Baha region. To calculate the sample size, the following formula was used:

$Sample Size = ((Z^2 xP(1-P))/e^2 ) /1+((Z^2 XP(1-P))/(e^2 N))$

where N is the total population (here, 506,866 as per 2020 data), P is the standard deviation, e is the margin of error (here, 5%), and z is the z-score (here, 1.96 at a 95% confidence level). With this in mind, the minimum sample size emerged as 384; however, the final sample size will be based on only the number of complete, reliable responses.

Data collection

To collect the data, the study utilizes a self-reported online questionnaire comprising three sections, the first of which includes a consent form that participants must complete before proceeding to the study questionnaire. The second section includes sociodemographic data gathering, such as gender, age, marital status, occupation, and level of education. The final section includes questions structured to assess the awareness and perception of participants regarding telemedicine. The questionnaire was distributed among participants through social media platforms, and data were collected through online methods, using an online Google Form in Arabic.

A pilot study with 20 participants was conducted, and the results were used to assess reliability and validity. A panel of three experts evaluated the questionnaire's validity by modifying the initial items to determine if they were suitable for evaluation. The subject matter experts were tasked with rating each item on a four-point scale based on its suitability and relevance, as shown in Table 1.

**Table 1:** Grading of the validation scores

Item	Score
Adequate (simple, relevant, and clear)	4
Adequate but needs minor revision	3
Needs major modification	2
Not so adequate (can be omitted)	1

Data analysis

The collected data were entered into SPSS version 26.0. Qualitative data was expressed as numbers and percentages, and a chi-squared test (χ2) was applied to test the relationship between variables. Multivariate regression analysis was conducted to assess independent predictors of good awareness and perception among studied participants. The odds ratio was calculated at a confidence interval (CI) of 95%. A p-value of <0.05 was considered statistically significant.

Ethical considerations

The study followed all the relevant ethical considerations, where the confidentiality and anonymity of the participant data are considered at all stages of the research, including gaining approval to proceed from the Al-Baha University research board during the first phase of data collection.

## Results

Study participants

Overall, 400 respondents responded to the survey; however, 41 participants were not from the Al-Baha region, so their data were not considered. Overall, data from 359 participants were considered. Table 2 represents the frequency and percentages of the demographic of the participants.

**Table 2:** Study participants’ basic descriptions

Variables	Frequency	Percentage
Age		
24-18	178	49.6
34-25	64	17.8
44-35	72	20.1
64-55	39	10.9
65 or over	6	1.7
Gender		
Female	212	59.1
Male	147	40.9
Nationality		
Non-Saudi	2	0.6
Saudi	357	99.4
Marital status		
Divorced	1	0.3
Married	160	44.6
Single	195	54.3
Widowed	3	0.8
Education level		
Bachelor's degree and above	249	69.4
High school	93	25.9
Middle school	11	3.1
Primary school	6	1.7
Occupation		
Student	153	42.6
Employee	113	31.5
Unemployed	65	18.1
Retired	26	7.2
Self-employed	1	0.3
Housewife	1	0.3
Area within the Al-Baha region		
Al-Baha	149	41.5
Al-Mandaq	85	23.7
Bani-Hassan	28	7.8
Baljurashi	24	6.7
Ghamd Al-zanad	19	5.3
Al-Gura	18	5
Al-Mekwa	13	3.6
Al-Hajra	13	3.6
Qelwah	10	2.8

As per the above table, most study participants were 18 to 24 years old representing 49.6% (178), with 20.1% (72) being between 35 and 44, and 17.8% (64) of participants being between 25 and 35 years. The results also show a predominant 59.1% (212) response from females, and 99.4% (357) being Saudi natives. Similarly, data revealed that 54.3% (195) of the study sample came from single participants, with 44.6% (160) of participants declaring that they were married. Regarding the education of the participants, it was found that 69.45% (249) of study participants had attained a bachelor's degree or higher, while 25.9% (93) had a high school education background. It was also found that 31.5% (113) of the participants were employees, while 42.6% (153) were students, and 18.1% (65) were unemployed. The participants were mostly from Al-Baha, while 23.75% (85) were from Al Mandaq, 7.8% (28) were from Bani Hassan, 6.7% (24) were from Baljurashi, which points to a high level of diversity in the respondents.

Awareness of telemedicine

To examine the awareness and perception of the participants, the frequency percentage for each of the questions was calculated with awareness and perception as the two criteria. The assessment of the responses can be seen in Table 3.

**Table 3:** Awareness among the general population about telemedicine

	Frequency	Percentage
Familiarity with the concept of telemedicine		
Slightly familiar	124	34.5
Moderately familiar	96	26.7
Not familiar at all	76	21.2
Very familiar	63	17.5
Ever used telemedicine services		
Yes	197	54.9
No	162	45.1
Type of telemedicine services used		
Online consultation with a healthcare professional	150	41.8
Video consulting with a healthcare specialist	78	21.7
Remote monitoring of a chronic condition	58	16.2
Never used	57	15.9
Follow-up on health status remotely	1	0.3
Mobile connection	1	0.3
Prescription re-disbursement	1	0.3
Monitor blood pressure readings	1	0.3
Contact through the Times program with home medicine	1	0.3
Aware of any of the services of communicative medicine provided in the community		
Yes	232	64.6
No	127	35.4

Regarding the participants' familiarity with telemedicine, 34.5% (124) of participants indicated that they were slightly familiar, while 26.7% (96) were moderately familiar with the concept, 21.25% (76) were not familiar, and 17.5% (63) were very familiar. Approximately 54.9% (197) have actual experience of using telemedicine, while 45.1% (162) have not. Regarding the type of telemedicine services used, it was found that most study participants, 41.8% (150), have used telemedicine services for video consulting with a healthcare specialist; 21.7% (78) have used video consulting with a healthcare specialist; and 16.2% (58) have used telemedicine for the remote monitoring of a chronic condition. The data also revealed that 64.6% (232) of study participants were already aware of the available communicative medicine services in their community, while 35.4% (127) had no awareness.

Perception of telemedicine

In terms of the perception of the participants regarding telemedicine as a concept, the outcome of each of the questions is presented in Table 4. Regarding the participants’ perception of the reason for using telemedicine services, the data shows that 31.92% (204) use it for time-saving purposes; followed by 31.3% (200), who use it for convenience; 20.97% (134) who use telemedicine services to access healthcare professionals; and 6.57% (42) using the facility for reasons of cost-effectiveness. Only 4.69% (30) had never used telemedicine services at the time of the data collection. While 26.145% (167) of participants expressed concerns relating to quality of care, 23.47% (150) indicated that privacy and security was an issue, 21.28% (136) mentioned limited availability, and 15.50% (99) had concerns relating to technical difficulties.

**Table 4:** Perception among the general population toward telemedicine

	Frequency	Percentage
Reasons for using telemedicine services		
Save time	204	31.92
Convenient	200	31.3
Access to healthcare professionals	134	20.97
Cost-effective	42	6.57
Never used	30	4.69
None	1	0.16
Repeat medication	1	0.16
Specific concerns or reservations about telemedicine services		
Quality of care	167	26.14
Privacy and security	150	23.47
Limited availability	136	21.28
Technical difficulties	99	15.50
Nothing	4	0.63
I have no concerns	1	0.16
I don’t know	1	0.16
The cost is sometimes high	1	0.16
No concerns	1	0.16
Information or education participants would like to receive regarding telemedicine services		
How to access telemedicine services	209	32.71
What type of services are available	185	28.95
How to ensure privacy and security	135	21.13
How to use the technology	104	16.28
All the above	2	0.31
I don’t know	1	0.16
Are you ready to try telemedicine to get a diagnosis and follow up on your health?		
Yes	287	79.9
I don't know	57	15.9
No	15	4.2
How telemedicine services could improve access to healthcare		
Reducing travel time and costs	138	38.4
Increased access to specialized care	83	23.1
Increased availability of healthcare professionals	78	21.7
Improved coordination of care	52	14.5
Never used	3	0.8
All of the below	1	0.9
No improvements	1	0.3

Furthermore, the data revealed that most participants, 32.71% (209), are willing to receive information about how to access telemedicine services; 28.95% (185) would like to receive information about the type of services available; while 21.13% (135) would be likely to accept information about how to ensure privacy and security in telemedicine services; and 16.28% (104) of participants would like to receive information relating to the use of the technology itself. Meanwhile, 79.9% (287) of respondents would use telemedicine to get a diagnosis and follow up on their health, while only 4.25% (15) would not. Approximately 38.4% (138) believe that telemedicine services could reduce travel time and costs, 23.1% (83) agree that it could increase access to specialized care, 21.7% (78) agree that it could improve the availability of healthcare professionals, and 14.5% (52) believe that these services could improve the coordination of care.

Variables affecting telemedicine awareness and perception

To examine the factors affecting telemedicine awareness and perception, the study examined the association between sociodemographic characteristics and the dimension of awareness and perception itself. To do so, the two most represented questions were chosen; namely, familiarity with the concept of telemedicine (awareness) and readiness to try telemedicine (perception). It was found that females and Al-Baha residents had a significantly higher percentage of people who were very familiar with the concept of telemedicine (p=<0.05) (Table 5).

**Table 5:** Association between sociodemographic characteristics and awareness N.B.: * = the X^2^ test was used

Variable	Familiarity with the concept of telemedicine				
	Not familiar at all	Slightly familiar	Moderately familiar	Very familiar	P value*
Variables					
Age					
24-18	33 (43.4)	62 (50)	49 (51)	34 (54)	0.222
34-25	12 (15.8)	15 (12.1)	20 (20.8)	17 (27)	
44-35	19 (25)	27 (21.8)	17 (17.7)	9 (14.3)	
64-55	10 (13.2)	18 (14.5)	9 (9.4)	2 (3.2)	
65 or over	2 (2.6)	2 (1.6)	1 (1)	1 (1.6)	
Gender					
Female	34 (44.7)	76 (61.3)	61 (63.5)	41 (65.1)	0.038
Male	42 (55.3)	48 (38.7)	35 (36.5)	22 (34.9)	
Nationality					
Non-Saudi	0 (0.0)	1 (0.8)	1 (1)	0 (0.0)	0.723
Saudi	76 (100)	123 (99.2)	95 (99)	63 (100)	
Marital status					
Divorced	0 (0.0)	1 (0.8)	0 (0.0)	0 (0.0)	0.555
Married	37 (48.7)	57 (46)	44 (45.8)	22 (34.9)	
Single	38 (50)	64 (51.6)	52 (54.2)	41 (65.1)	
Widowed	1 (1.3)	2 (1.6)	0 (0.0)	0 (0.0)	
Education level					
Bachelor's degree and above	47 (61.8)	92 (74.2)	71 (74)	39 (61.9)	0.11
High school	21 (27.6)	30 (24.2)	21 (21.9)	21 (33.3)	
Middle school	6 (7.9)	1 (0.8)	3 (3.1)	1 (1.6)	
Primary school	2 (2.6)	1 (0.8)	1 (1)	2 (3.2)	
Occupation					
Student	25 (32.9)	48 (38.7)	48 (50)	32 (50.8)	0.333
Employee	26 (34.2)	41 (33.1)	27 (28.1)	19 (30.2)	
Unemployed	15 (19.7)	26 (21)	14 (14.6)	10 (15.9)	
Retired	9 (11.8)	9 (7.3)	6 (6.3)	2 (3.2)	
Self-employed	1 (1.3)	0 (0.0)	0 (0.0)	0 (0.0)	
Housewife	0 (0.0)	0 (0.0)	1 (1)	0 (0.0)	
Area within the Al-Baha region					
Al-Baha	54 (43.5)	42 (43.8)	28 (36.8)	25 (39.7)	0.001
Al-Mandaq	35 (28.2)	21 (21.9)	14 (18.4)	15 (23.8)	
Bani-Hassan	6 (4.8)	5 (5.2)	2 (2.6)	0 (0.0)	
Baljurashi	2 (1.6)	8 (8.3)	4 (5.3)	4 (6.3)	
Ghamd Al-Zanad		2 (2.1)	9 (11.8)	1 (1.6)	
Al-Gura	1 (0.8)	7 (7.3)	5 (6.6)	4 (6.3)	
Al-Mekwa	8 (6.5)	4 (4.2)	13 (17.1)	5 (7.9)	
Al-Hajra	6 (4.8)	6 (6.3)	1 (1.3)	6 (9.5)	
Qelwah	6 (4.8)	1 (1)	0 (0.0)	3 (4.8)	

However, young participants (18-24 years), females, and students had a significantly higher percentage of those ready to try telemedicine to get a diagnosis and follow up on their health (p=<0.05) (Table 6).

**Table 6:** Association between sociodemographic characteristics and perception N.B.: * = the X^2^ test was used

Variables	Are you ready to try telemedicine to get a diagnosis and follow up on your health?			P value*
	I don't know	No	Yes	
Age				
24-18	36 (63.2)	8 (53.3)	134 (46.7)	0.007
34-25	2 (3.5)	1 (6.7)	61 (21.3)	
44-35	15 (28.3)	5 (33.3)	52 (18.1)	
64-55	4 (7)	0 (0.0)	35 (12.2)	
65 or over	0 (0.0)	1 (6.7)	5 (1.7)	
Gender				
Female	44 (77.2)	8 (53.3)	160 (55.7)	0.01
Male	13 (22.8)	7 (46.7)	127 (44.2)	
Nationality				
Non-Saudi	0 (0.0)	0 (0.0)	2 (0.7)	0.777
Saudi	57 (100)	15 (100)	285 (99.3)	
Marital status				
Divorced	0 (0.0)	0 (0.0)	1 (0.3)	0.154
Married	22 (38.6)	8 (53.3)	130 (45.3)	
Single	34 (59.6)	6 (40)	155 (54)	
Widowed	1 (1.8)	1 (6.7)	1 (0.3)	
Education level				
Bachelor's degree and above	33 (57.9)	8 (53.3)	208 (72.5)	0.235
High school	20 (35.1)	6 (40)	67 (23.3)	
Middle school	2 (3.5)	1 (6.7)	8 (2.8)	
Primary school	2 (3.5)	0 (0.0)	4 (1.4)	
Occupation				
Student	31 (54.4)	4 (26.7)	118 (41.1)	0.004
Employee	6 (10.5 )	4 (26.7)	103 (35.9)	
Unemployed	15 (26.3)	6 (40)	44 (15.3)	
Retired	4 (7)	1 (6.7)	21 (7.3)	
Self-employed	1 (1.8)	0 (0.0)	0 (0.0)	
Housewife	0 (0.0)	0 (0.0)	1 (0.3)	
Area within the Al-Baha region				
Al-Baha	26 (45.6)	7 (46.7)	116 (40.4)	0.461
Al-Mandaq	13 (22.8)	2 (13.3)	70 (24.4)	
Bani-Hassan	6 (10.5)	2 (13.3)	20 (7)	
Baljurashi	3 (5.3)	2 (13.3)	19 (6.6)	
Ghamd Al-Zanad	1 (1.8)	2 (13.3)	16 (5.6)	
Al-Gura	1 (1.8)	0 (0.0)	17 (5.9)	
Al-Mekwa	1 (1.8)	0 (0.0)	12 (4.2)	
Al-Hajra	2 (3.5)	0 (0.0)	11 (3.8)	
Qelwah	4 (7)	0 (0.0)	6 (2.1)	

In the multivariate logistic regression analysis, gender and residence were not found to be significant predictors of awareness of telemedicine among participants, with gender exhibiting a regression coefficient (B) of 0.28, a Wald statistic of 1.44, p-value of 0.23, and an odds ratio (OR) of 1.23 (95% CI: 0.83-2.1). Residence was similarly nonsignificant, with B = 0.23, Wald statistic of 0.12, p-value of 0.725, and OR of 1.26 (95% CI: 0.34-4.68). In assessing the perception of telemedicine, gender was also not statistically significant (B = 0.32, Wald statistic = 0.79, p-value = 0.372, OR = 0.72, 95% CI: 0.35-1.47). However, occupation emerged as a significant predictor for the perception of telemedicine with B = 1.4, Wald statistic of 8.97, P value of 0.003, and an OR of 4.08 (95% CI: 1.26-10.26), indicating a substantial likelihood of positive perception in certain occupational groups (Table 7).

**Table 7:** Multivariate logistic regression analysis of the independent predictors of good awareness and perception among studied participants

Variable	B	Wald	P value	Odds ratio (CI:95%)
Awareness				
Gender	0.28	1.44	0.23	1.23 (0.83-2.1)
Residence	0.23	0.12	0.725	1.26 (0.34-4.68)
Perception				
Gender	0.32	0.79	0.372	0.72 (0.35-1.47)
Occupation	1.4	8.97	0.003	4.08 (1.26-.10.26)

## Discussion

It is crucial to study the perception and awareness regarding telemedicine as a way to better inform the future implementation of rollout strategies. This study revealed a low level of familiarity regarding telemedicine among the general population, with nearly half the study sample having never used the service. The most common use of telemedicine among those respondents who have had experience with the technology is online consulting with a healthcare professional, the remote monitoring of chronic conditions, and video consulting with a healthcare specialist. Despite the lack of use in the sample, the study still revealed a generally positive perception, with participants believing that telemedicine helps reduce travel time and leads to improved coordination of care, increased access to specialized care, and increased availability of healthcare professionals.

These outcomes mirror the findings by Albarrak et al. [8], Alnajrani et al. [9], and Mubaraki et al. [10], who reported low-level knowledge and a positive level of perception among different sections of the population in Saudi Arabia for a range of potential advantages, such as improved coordination of care, increased access to specialized care, increased availability of healthcare professionals, and reduced travel time and costs. These benefits would facilitate the reduction of unnecessary visits to health practitioners and the earlier detection of illnesses. Generally, telemedicine has several benefits for the healthcare system, including quick access to reliable medical information, increased access to higher-quality healthcare services in locations with limited resources, and lower healthcare costs [17]. Indeed, several telehealth technologies have already been implemented to enhance healthcare quality. The effective implementation of a Telehealth Emergency SystemImplications is also required [18].

Interestingly, the multivariate logistic regression analysis of our data elucidates the differential impact of demographic and occupational variables on the awareness and perception of telemedicine. Gender and residence did not emerge as significant predictors for awareness, nor did gender for perception. This indicates that these demographic factors may not play a pivotal role in telemedicine adoption, as supported by a study assessing demographic influences on telemedicine use​​ [19]. However, occupation showed a significant association with perception, aligning with findings that clinician attitudes toward telehealth vary with specialty, impacting utilization rates​​ [20]. Individuals within certain occupational categories were more likely to have a positive perception of telemedicine. This highlights the need for public health strategies to consider the influence of professional roles and the potential for tailored interventions, as occupational perspectives are crucial in shaping telemedicine perception and usage [20]. Additionally, addressing the identified barriers such as technical challenges and resistance to change, as well as enhancing training and policy interventions, is essential for widespread telemedicine adoption​​ [21].

Furthermore, the study explores barriers to the mass utilization of telemedicine services, such as limited availability, privacy and security concerns, and perceived quality of care. Al-Hazmi et al. [12] and Al-Samarraie et al. [13] explored similar barriers, with the overall findings of these studies suggesting that the implementation of quality education would be highly beneficial for improving healthcare services. The significant role of occupation in shaping perception, as revealed by our study, suggests that such educational interventions should be particularly targeted at professional groups who may not yet perceive the full range of telemedicine benefits, thereby addressing one of the key barriers to telemedicine adoption.

Study

The study has provided valuable insight into the general population's perception and awareness of telemedicine services. These findings are crucial for the general population and health practitioners to better understand the role that telemedicine can play in the country and its beneficial aspects. The findings will also assist health authorities in investing more heavily in the quality of educational literature regarding access to telemedicine services and the use of the technology itself. Moreover, potential strategies can be implemented to mitigate the major risks; namely, limited availability, privacy and security, and quality of care. Overall, the study provides a theoretical contribution to the literature regarding the utilization of telemedicine among the general public in the region.

Limitations

Although the study has presented valuable data regarding the awareness and perception of the general public, there are limitations. First, the study does not utilize the standard scale measurement for assessing awareness and perception. Rather, the convenience sampling technique was used, which can result in sampling error and respondent biases. Moreover, study findings can be limited because of the time effect, as population views and perceptions are likely to change with time.

Future directions

This study provides direction for future studies, which could further explore the perception and knowledge of telemedicine services among specific segments of the population, such as older people or women. Similarly, future studies could be structured to compare telemedicine services among different regions, including Al-Baha. Moreover, researchers could also explore the strategic framework for telemedicine provision services in the region by exploring the risks and benefits associated with the implementation of existing strategies.

## Conclusions

This study found that almost half of the participants have never used telemedicine services, whereas the most common use is online consultation with a healthcare professional. The main concerns of the participants toward telemedicine were the limited availability, privacy and security, and quality of care. However, the majority were ready to try telemedicine to get a diagnosis and follow up on their health indicating a positive perception. There is a need to implement strategies to strengthen the security and privacy of telemedicine services. Additionally, there is a need to educate the public about the value and effectiveness of telemedicine.
